# Co-occurrence of Anti-N-Methyl-D-Aspartate Receptor Encephalitis and Anti-myelin Oligodendrocyte Glycoprotein Inflammatory Demyelinating Diseases: A Clinical Phenomenon to Be Taken Seriously

**DOI:** 10.3389/fneur.2019.01271

**Published:** 2019-12-04

**Authors:** Yijun Ren, Xiqian Chen, Qiang He, Renchun Wang, Wei Lu

**Affiliations:** ^1^Department of Neurology, The Second Xiangya Hospital, Central South University, Changsha, China; ^2^The Second Clinical Medicine School of Lanzhou University, Lanzhou, China

**Keywords:** autoimmune encephalitis, N-methyl-D-aspartate (NMDA), demyelinating diseases, myelin oligodendrocyte glycoprotein (MOG), immunotherapy

## Abstract

**Background:** Anti-N-methyl-D-aspartate receptor (NMDAR) immunoglobulin G antibodies which exist on myelin sheaths, composed of oligodendrocytes, especially target GluN1 subunits and are highly characteristic of anti-NMDAR encephalitis which is a newly recognized autoimmune encephalitis (AE) characterized by psychiatric symptoms, behavioral abnormalities, seizures, cognitive impairment and other clinical symptoms. Myelin oligodendrocyte glycoprotein (MOG) is a type of protein which is expressed on the surface of oligodendrocytes and myelin in the central nervous system. Anti-MOG antibodies cause demyelination. In some rare reported cases, these two types of antibodies have been found to co-exist, but the underlying mechanisms remain unknown.

**Case presentation:** Here we report cases of 4 inpatients (median age 31.5 years, age range 27–43 years) from The Second Xiangya Hospital of Central South University between March 2018 and April 2019. Two of the cases were first diagnosed as anti-NMDAR encephalitis and had developed visual impairments in the course of the dosage reduction during corticosteroid therapy. They were found at the time, to have anti-MOG antibody-positive CSF and/or serum. Another patient was diagnosed with anti-MOG inflammatory demyelinating diseases (IDDs) when he tested double positive for both anti-NMDAR and anti-MOG antibodies early in the course of his illness. Over the course of the dosage reduction during corticosteroid therapy, his symptoms deteriorated; however, anti-MOG antibody levels elevated while anti-NDMAR antibody levels remained low. The other patient had initially developed psychiatric symptoms and limb weakness. She was also double positive for anti-NMDAR and anti-MOG antibodies early in the course of her illness. However, over the course of the dosage reduction during corticosteroid therapy, her symptoms worsened and levels of both antibodies elevated.

**Conclusion:** Anti-NMDAR and anti-MOG antibodies may coexist in rare cases. In addition, anti-NMDAR encephalitis and anti-MOG inflammatory demyelinating diseases may occur either simultaneously or in succession. Thus, when a patient is diagnosed with either of these two diseases, but exhibits symptoms of the other disease, the possibility of co-occurrence with both these diseases should be considered and the appropriate antibodies should be accurately detected to enable prompt selection of appropriate treatments by the physicians.

## Introduction

Anti-N-methyl-D-aspartate receptor (NMDAR) encephalitis is a severe, but treatable autoimmune disorder with clinical manifestations of psychiatric and neurologic symptoms. It is often accompanied by a teratoma or other neoplasms, especially in female patients (1–6). Anti-NMDAR antibody-positive cerebrospinal fluid (CSF) or serum are characteristic, of the disease (5, 6). Myelin oligodendrocyte glycoprotein (MOG) is a type of protein which is expressed on the surface of oligodendrocytes and myelin in the central nervous system (CNS) (7). Antibodies to MOG can be detected in patients with inflammatory demyelinating diseases (IDDs) of the CNS (8). The international consensus is now that, anti-MOG antibodies result in demyelinating diseases, of the neuromyelitis optical spectrum disorders (NMOSD) (7, 9, 10). The pathogenic mechanisms of these two diseases were once believed to be entirely different, but several cases have recently reported the coexistence of anti-NMDAR and anti-MOG antibodies (3, 11–13). However, these consisted of individual cases or small sample reports, and no systematic review of large-scale samples has summarized, to date, the characteristic features of the coexistence of anti-NDMAR encephalitis and anti-MOG IDDs.

The purpose of this report is to discuss the possible mechanisms for the coexistence of multiple autoimmune antibodies, which leads to different autoimmune diseases, by comparing patients with partially similar clinical presentations.

## Materials and Methods

### Patient Inclusion

This study was approved by the Ethics Committee of the Second Xiangya Hospital of Central South University. In this retrospective observational study, we analyzed four inpatients between March 2018 and April 2019, who were double positive for anti-NMDAR and anti-MOG antibodies in serum and/or cerebrospinal fluid.

### Antibody Identification

The antibodies panel included anti-NMDAR, anti-GABABR, anti-AMPA1, anti-AMPA2, anti-CASPR2, anti-LGI1, anti-AQP-4, and anti-MOG. Antibodies testing were done through cell-based assays (BCA) in the Guangzhou King Med Center for Clinical Laboratory. Following the guidelines of Guangzhou King Med Center for Clinical Laboratory, the antibody cut-off level was 1:32, and full-length human antigenic substrates were used.

## Results

Here we describe the cases of four inpatients at the Second Xiangya Hospital of Central South University between March 2018 and April 2019, who were either seropositive and/or CSF-positive for anti-NMDAR and anti-MOG antibodies. Patient 1 and 2 had symptoms typical of autoimmune encephalitis, including cephalalgia, speech disorder, and decreased consciousness, each of which meets the diagnostic criteria for anti-NMDAR encephalitis (see Table 1) (5). They were found to be anti-NMDAR antibody positive. Over the course of dosage reduction during corticosteroid treatment, these two patients developed visual impairments and were found to be anti-MOG antibody positive. Patient 3 developed dizziness, double vision, and weakness of the right limb but no visual impairment. He was found to be simultaneously anti-NMDAR and anti-MOG antibody-positive (Figures 2A,C). Based on the combination of clinical features and laboratory evidence, the patient was diagnosed with an anti-MOG inflammatory demyelinating disease, though the anti-NMDAR antibody titer was too low to establish a definitive diagnosis of anti-NMDAR encephalitis. Over the course of his immunosuppressive treatment, he developed visual impairment and his anti-MOG antibody titer increased (Figures 2D,E) (his anti-NDMAR antibody titer remained low simultaneously, Figure 2B). Patient 4 first developed cephalalgia, psychiatric symptoms, weakness and numbness of the limbs early in the course of her illness. She was found, at that time, to be double positive for anti-NMDAR and anti-MOG antibodies in the CSF and serum. Considering the clinical symptoms, laboratory findings and imaging findings, she was diagnosed with anti-MOG-positive NMOSD with low titer of anti-NMDAR antibodies. She also developed visual impairments over the course of dosage reduction during corticosteroid therapy, accompanied with increase in the anti-NMDAR and anti-MOG antibody titers. She was finally diagnosed with anti-MOG-positive NMOSD and anti-NMDAR encephalitis.

**Table 1:** Clinical characteristic laboratory data and treatments of cases in first and relapse episode.

**Characteristic**	**Patient 1**	**Patient 2**	**Patient 3**	**Patient 4**
Initial Symptoms	Alalia, cephalalgia, numbness of right upper limb	Weakness and numbness of left upper limb, disturbance of consciousness	Dizziness, double vision, weakness of right limb	Cephalalgia, psychiatric symptoms, weakness, and numbness of limbs
**LABORATORY DATA OF INITIAL EPISODE**				
**CSF Findings**				
CSFP, mmH^2^O	190	280	170	150
CSF WBC*10^6^/L	8	240	30	88
CSF protein, mg/dL	166.4	250.6	293.3	444.52
CSF/Serum anti-NMDAR-ab	1:32 pos/1:10 pos	1:32 pos/1:10 pos	1:10 pos/Na	1:10 pos/Neg
CSF/serum antibodies to AMPA1, AMPA2, CASPR2, LGI1, GABAR	Neg	Neg	Neg	Neg
CSF/Serum anti-MOG-ab^a^	Na/Na	Na/Na	1:1 pos/Na	1:10 pos/1:32 pos
Other Findings	TPO-ab pos	Na	anti-HSV IgG pos	Na
Imaging findings of initial episode	Abnormal swelling of the left hippocampus with high signal in T2/flair (Figure 1A)	MRI revealed lesions that were high signal on T1WI and T2WI in callosum and around the ventricle (Figure 1C)	Extensive intracranial lesions (Figure 1E)	High signal on T1WI/T2WI in midbrain, high signal on T2 fat-suppression sequence in C7-T1/T7-T12 spinal cord plane
Treatments with immunosuppressant	High-dose intravenous corticosteroids, orally corticosteroids treatment (initial 60 mg/d and decreased 5 mg every 2 weeks)	IVIG, High-dose intravenous corticosteroids, natalizumab infusions (600 mg), MMF	High-dose intravenous corticosteroids, orally corticosteroids treatment (initial 60 mg/d and decreased 5 mg every 2 weeks), MMF	High-dose intravenous corticosteroids, orally corticosteroids treatment (initial 60 mg/d and decreased 5 mg every 2 weeks), AZA
Time since symptoms relieved to elapse episode, mo	7	0.5	12	6
Dosage of immunosuppressor	Drug withdrawal	MMF (500 mg/d)	Irregularly medication	Corticosteroids(30 mg/d), AZA(100 mg/d)
Relapse symptoms	Blurred vision in right eye	Acute and progressive diminution of vision	Dizziness, blurred vision	Blurred vision, diplopia
**LABORATORY DATA OF RELAPSE EPISODE**				
CSFP, mmH2O	140	200	190	140
CSF WBC*106/L	8	4	4	40
CSF protein, mg/dL	235.1	321.8	166.87	373.18
CSF/Serum anti-NMDAR-ab	1:32 pos/1:32 pos	1:32 pos/Neg	1:10 pos/Na	1:32 pos/1:10 pos
CSF/Serum anti-MOG-ab	1:10 pos/neg	1:10 pos/1:32 pos	1:100 pos/1:32 pos	1:100 pos/1:100 pos
Other Findings	Na	Na	Na	Na
Imaging findings of relapse episode	High signal in T2/flair in both right and hippocampal region (Figure 1B)	High signal on T1WI and T2WI in centrum semiovale; enlargement of the left optical nerve (Figure 1D)	High signal on T1WI/T2WI/T2Flair in posterior horn of lateral ventricle and right cerebellar hemisphere (Figure 1F)	New high signal lesions on T1WI/T2WI in right pons and aqueduct of sylvius, Spinal cord lesions are as mentioned before
Treatment of relapse episode	Treatment refusal	orally corticosteroids treatment	Orally corticosteroids treatment, AZA	High-dose intravenous corticosteroids, orally corticosteroids treatment (initial 60 mg/d and decreased 5 mg every 2 weeks), AZA(150 mg/d)

*MOG, myelin oligodendrocyte glycoprotein; AQP4, aquaporin 4; NMDAR, N-methyl-D-aspartate receptor; CASPR2, contactin associated protein 2; LGI1, leucine-rich glioma-inactivated 1; AMPAR, anti-alpha-amino-3-hydroxy-5-methyl-4-isoxazolepropionic acid receptor; GABAR, anti-γ-aminobutyric acid receptor; CSF, cerebrospinal fluid; IVIG, intravenous immunoglobulin; AZA, azathioprine; MMF, mycofenolate mofetil; WBC, white blood cell; TPO-ab, thyroid peroxidase antibody; Neg, negative; pos, positive; Na, not applicable*.

a *Serum MOG-IgG cut-off 1:32; assay: CBA, Guangzhou King Med Center for Clinical Laboratory; antigen: full-length human MOG (14)*.

**Figure 1:**
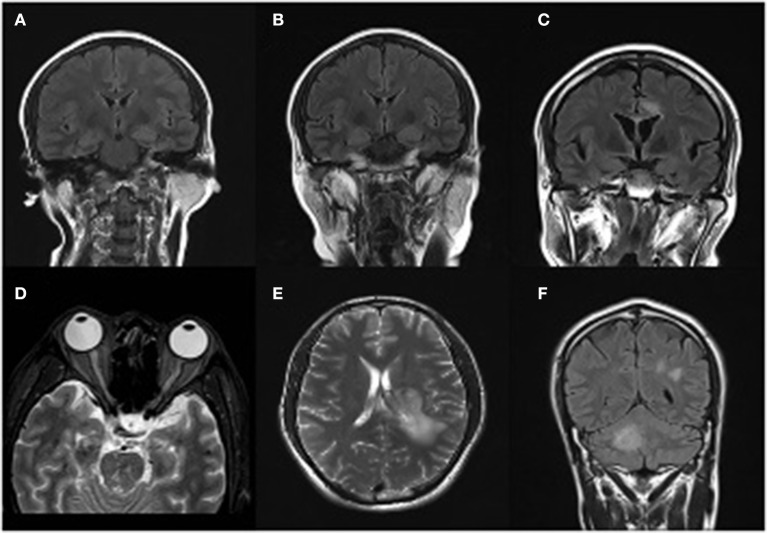
Magnetic resonance imaging presentations. Case 1 **(A)** T2/flair image reveals swelling of the left hippocampus in March 2018. **(B)** Swelling of the double-side hippocampus during the relapse episode in October 2018. Case 2 **(C)** MRI revealed lesions that were high signal on T1WI and T2WI in callosum and around the ventricle in October 2018. **(D)** An obvious enlargement of the left optical nerve, a diagnostic evidence of Neuromyelitis Optica Spectrum Disorders (NMOSD), during his second episode in November 2018. Case3 **(E)** MRI revealed lesions that were high signal on T1WI and T2WI in the left hemisphere in 29th March 2018. **(F)** High signal on T1WI/T2WI/T2Flair in posterior horn of lateral ventricle and right cerebellar hemisphere.

**Figure 2:**
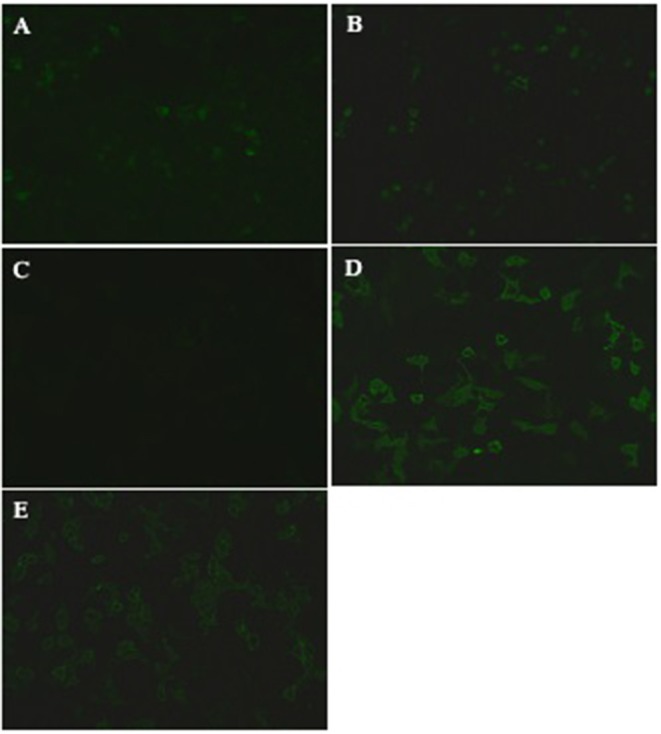
Demonstration of anti-MOG and NMDAR antibodies of a representative case (patient 3). Positive anti-MOG and NMDAR antibodies validated by transfected cell based indirect immune-fluorescence test. **(A)** CSF was NMDAR antibodies positive (titer, 1:10) in March 2018. **(B)** CSF was still NMDAR antibodies positive (titer, 1:10) in March 2019. **(C)** CSF was anti-MOG positive (titer, 1:1) in March 2018. **(D)** CSF (titer, 1:100) and **(E)** serum (titer, 1:32) were both anti-MOG positive in March 2019.

All of the four patient were seronegative and CSF-negative for anti-GABABR, anti-AMPA1, anti-AMPA2, anti-CASPR2, and anti-LGI1 antibodies, which are antibodies known to cause autoimmune encephalitis (4, 5). In addition, they were also anti-AQP-4 antibody-negative in serum and CSF. Detailed clinical information is summarized in the Table 1.

These patients received different types of immunosuppressive treatments, the corticosteroid therapy being commonly administered to all patients; consequently, they were relieved of their clinical symptoms. However, on dosage reduction or total suppression of immunosuppressant therapy, all the patients developed visual impairments, thereby, needing hospitalization again. On assessment, anti-MOG and anti-NMDAR antibody titers in the CSF and/or serum showed altered levels.

Two of the 4 patients were male, with a median onset age of 31.5 years (age range 27–43 years). The median time from initial symptoms until the onset of visual impairment was 6.5 months (range 0.5–12 months). Patients 1, 3, and 4 developed visual impairments on dosage reduction (or withdrawal) of corticosteroids, and patient 2 additionally received a Mycophenolate Mofetil treatment.

## Discussion

Anti-NMDAR encephalitis has recently been clinically recognized, and is reported to be associated with the presence of specific CSF IgG antibodies to NMDA receptors, especially against GluN1 subunits (4). Anti-NMDAR encephalitis commonly causes psychiatric symptoms, behavioral abnormalities, seizures, speech disorder, cognitive impairment and decreased consciousness (1, 2, 4–6). It has also been reported to sometimes co-occur with a teratoma, especially in female patients, or a intracranial viral infection (15). Neuromyelitis optical (NMO) was once believed to be associated with the presence of autoantibodies to aquaporin-4 (AQP-4) (16). However, further research revealed that 10–20% of patients with NMO were negative for AQP4-IgG but positive for MOG antibodies (8, 9, 17). MOG-IgG-related NMO is now widely recognized as a specific disease (10). In a cohort study of 50 patients, the sex ratio was found to be 1:2.8 (M:F) and the median age was 31 years (age range 6–70 years) (8). These data are characteristic to severe visual impairment or functional blindness, and markedly impaired ambulation. Responses to intravenous methylprednisolone and long-term immunosuppression treatment were satisfactory (2, 4, 6, 8, 17, 18).

The coexistence of MOG and NMDAR antibodies has so far only rarely been reported. Titulaer et al. (3) conducted a retrospective analysis of 691 cases of anti-NMDAR encephalitis, including 23 patients who were diagnosed with anti-NMDAR encephalitis with comorbid symptoms of demyelination. Among them, nine patients had anti-MOG antibodies with similar serological findings to the four cases described in this report. Zhou et al. (12) reported the case of a 31 year-old man whose initial symptoms were fever, headache and seizures, alongside numbness, neuritis and unsteady walking during subsequent episodes. In this case, both anti-NMDAR and anti-MOG antibodies were simultaneously detected, however, researchers believe that the diagnosis of MOG-IDD is definite, while that of AE remains controversial. This is due to the fact that, according to them, NMDAR antibodies were not present when the patient showed typical AIE symptoms. However, Sarigecili et al. (13) reported a case of co-occurrence of antibodies against both MOG and NMDAR in a child, and authors believed that multiple seropositivity may be more frequent than is reported. Four cases in our report had multiple clinical features of central nervous system autoimmune diseases in the first episode and the disease remission was adequate in response to immunosuppressive therapy, but patients subsequently developed severe visual impairments.

Here, we report four cases of patients who are double positive for both anti-NMDAR and anti-MOG antibodies. The clinical course of two of these cases matched previous reports from the literature, suggesting that patients contract anti-NMDAR encephalitis followed by anti-MOG IDDs. However, anti-MOG IDDS could also occur first, followed by anti-NMDAR encephalitis, and a certain type of pathogenic antibody may be critical in the course of disease. In addition, anti-NMDAR encephalitis and anti-MOG IDDs could also occur simultaneously (3, 12, 13). These findings suggest that, when a patient is diagnosed with either of these two diseases, the potential co-occurrence of the other disease should be considered by the medical practitioners.

The coexistence of MOG-IgG and anti-NMDAR-IgG may result, firstly, from autoimmune disorders targeting oligodendrocytes. MOG is a type of protein which is expressed on the surface of oligodendrocytes and myelin in the central nervous system (7). Additional studies have pointed out that functional NMDARs exist on oligodendrocytes (12, 19, 20). Thus, these two types of autoantigens may be present on the surface of oligodendrocytes simultaneously (7). During the pathological process of autoimmunity, the immune cells may erroneously attack MOG and NMDAR autoantigens, which are in the same location, and produce anti-NMDAR and anti-MOG antibodies in CSF and serum. These autoimmune antibodies may eventually lead to the onset of disease. However, the rate of co-occurrence of autoimmune encephalitis and a demyelinating disease remains unclear, to date (3). Secondly, multiple seropositivity may also be attributed to immune reconstitution. It is widely known that different types of immunotherapies (including corticosteroid treatment) may influence immunological status. On dosage reduction or withdrawal of immunotherapy, the immune system will recover from immunosuppression (21) and rebuild itself; thus causing immune cells to attack autoantigens and lead to inflammatory CNS responses (22–24). Some researchers have found that AIDS patients who are immunodeficient may relapse or develop other complications during the course of combination antiretroviral therapy (25, 26). This is termed immune reconstitution inflammatory syndrome (IRIS). Researchers have attributed IRIS to immune reconstitution (i.e., changes in CD4+ T cell counts) (21, 23). Similarly, relapses and new clinical syndromes caused by new-onset autoimmune antibodies or increase in the titers, thereof, could result during dosage reduction or withdrawal of corticosteroid therapy.

Thus, we insist that anti-NMDAR and anti-MOG antibodies can exist simultaneously, sometimes even when the initial symptoms of patients are primarily caused by one of these two antibodies. Regardless of whether a patient first presents with clinical symptoms of anti-NMDAR encephalitis or anti-MOG IDDs, if a patient develops new impairments during the course of immunity-regulating therapies, the potential co-occurrence of these two types of diseases must be considered. If a patient is found double positive for these two types of antibodies, especially when new symptoms develop during immunotherapeutic treatment, the immunomodulatory therapy protocols should be expeditiously developed, and the most suitable treatment protocols should be carefully selected in advance.

## Conclusions

We believe that anti-MOG and anti-NMDAR antibodies may coexist either simultaneously or in succession. As such, anti-NMDAR encephalitis and anti-MOG IDDs could occur simultaneously or successively. Therefore, neurologists should pay close attention to these conditions and choose appropriate treatment strategies.

## Data Availability Statement

All datasets generated for this study are included in the article/supplementary material.

## Ethics Statement

This study was approved by the Ethics Committee of the Second Xiangya Hospital of Central South University. In this retrospective observational study, we analyzed four inpatients between March 2018 and April 2019, who were double positive for anti-NMDAR and anti-MOG antibodies in serum and/or cerebrospinal fluid.

## Author Contributions

WL designed the study. YR, XC, QH, and RW performed the clinical materials collections. YR did the literature search and wrote the paper. XC, QH, and WL reviewed and edited the manuscript. WL takes responsibility for the integrity of the work from its inception to publishing. All authors read and approved the manuscript.

### Conflict of Interest

The authors declare that the research was conducted in the absence of any commercial or financial relationships that could be construed as a potential conflict of interest.
